# Domestic dogs are mammalian reservoirs for the emerging zoonosis flea-borne spotted fever, caused by *Rickettsia felis*

**DOI:** 10.1038/s41598-020-61122-y

**Published:** 2020-03-05

**Authors:** Dinh Ng-Nguyen, Sze-Fui Hii, Minh-Trang Thi Hoang, Van-Anh Thi Nguyen, Robert Rees, John Stenos, Rebecca Justine Traub

**Affiliations:** 1 Tay Nguyen University, Buon Ma Thuot, Dak Lak Vietnam; 2 Australian Rickettsial Reference Laboratory, Geelong, Victoria Australia; 3 Buon Ma Thuot University, Buon Ma Thuot, Dak Lak Vietnam; 4 The University of Melbourne, Parkville, Victoria Australia; 5 Bayer Australia, Pymble, New South Wales Australia

**Keywords:** Bacterial host response, Parasite host response

## Abstract

*Rickettsia felis* is an obligate intracellular bacterium that is being increasingly recognized as an etiological agent of human rickettsial disease globally. The agent is transmitted through the bite of an infected vector, the cat flea, *Ctenocephalides felis*, however there is to date, no consensus on the pathogen’s vertebrate reservoir, required for the maintenance of this agent in nature. This study for the first time, demonstrates the role of the domestic dog (*Canis familiaris*) as a vertebrate reservoir of *R. felis*. The ability of dogs to sustain prolonged periods of rickettsemia, ability to remain asymptomatically infected with normal haematological parameters and ability to act as biological vehicles for the horizontal transmission of *R. felis* between infected and uninfected fleas provides indication of their status as a mammalian reservoir of this emerging zoonosis.

## Introduction

Rickettsiae are obligate intracellular alpha-proteobacteria, maintained in nature through arthropod vectors and the vertebrate hosts they infect. Vertebrate hosts capable of developing rickettsemias, termed reservoir hosts, in turn, allow new lines of arthropod vectors to acquire infection. Except for epidemic typhus caused by *Rickettsia prowazekii* and transmitted by the human body louse, humans represent accidental or end-stage hosts for these agents and play no role in their life cycle.

*Rickettsia felis* URRWXCal2 is being increasingly implicated as an important cause of non-specific febrile illness in humans globally^1–3^. When symptoms manifest, they most often present as undifferentiated flu-like illness (fever, myalgia, and headache) but occasionally may progress to more severe manifestations which may include fever, ‘rash’ and neurological symptoms^2,4,5^. Various arthropods such as fleas, ticks, mites, and mosquitoes have been found to be associated with *R. felis*^6–11^, but of these, the cat flea, *Ctenocephalides felis felis* is the only confirmed biological vector for *R. felis* isolate URRWXCal2^12–14^, capable of vertically transmitting the agent for up to 12 generations without a blood meal^15^. However to date, the range of natural vertebrate reservoir for the pathogen remains unknown^16^. Recently, field surveys have demonstrated cats, dogs, opossums, raccoons and rodents to be seropositive or PCR-positive for *R. felis* DNA^12,17–21^, suggesting that these vertebrate species may also act as potential mammalian reservoirs for *R. felis*

Our understanding of horizontal transmission mechanisms of *R. felis* in cat fleas feeding on vertebrate hosts is scarce and incomplete^12^. The research of Wedincamp and Foil (2000)^22^ was the first to demonstrate the presence of *R. felis* URRWXCal2 DNA in the blood of 5 of 16 cats two months following being fed on by *R. felis*-positive fleas. However, they failed to demonstrate transmission of *R. felis* to the progeny of *C. felis* fed on these *R. felis*-positive cats^15^. The detection of *R. felis* URRWXCal2 DNA in 9% of healthy pound dogs in Australia^23^ and 11% of healthy community dogs in Cambodia^24^ strongly suggest the presence of a domestic cycle for *R. felis*, with domestic dogs being the likely reservoir hosts.

The primary aim of this study was to test the hypothesis that horizontal transmission of *R. felis* occurs from infected to uninfected fleas and their progeny through feeding on dogs with a naturally acquired rickettsemia, a feature that is central to the role of reservoir hosts. We also describe the clinical signs, hematological indices and immunological responses of *R. felis*-infected dogs.

## Results

All nine puppies were tested and found to be PCR- and seronegative to *R. felis* before inclusion in the study.

### *R. felis* needle-inoculation of dogs

After subcutaneous inoculation of *R. felis*, DNA of *R. felis* was detected in the blood of Dogs A and B on day eight post inoculation (pi). Antibodies against *R. felis* were detected in Dogs A and B on day three pi at titers of 1:128. Dogs A and B were positive for *R. felis* by culture on day eight pi.

*R. felis*-negative (RfNEG) fleas placed on Dogs A and B on day 62 pi were positive for *R. felis* by PCR and culture on Day 68 pi. Emerged nymphs sourced from eggs collected on day 68 were PCR and culture-positive for *R. felis*. The second new set of RfNEG fleas on Dogs A and B were *R. felis* positive by PCR and culture three days following their placement.

*R. felis* was detected in the blood of Dogs C and D seven- and five- days pi, respectively by PCR, but not by culture. Antibodies to *R. felis* were detected in Dog C on day three and Dog D on day four pi, at titers of 1: 256 and 1:128, respectively. Duplicate pools, each consisting of five RfNEG fleas that fed on Dogs C and D were positive for *R. felis* by PCR and culture on Day 12 pi. Emerged nymphs sourced from eggs collected on day 12 were PCR and culture-positive for *R. felis*.

A new set of 40 RfNEG fleas placed on Dogs C and D on day 15 pi were PCR and culture positive three-days post-feeding (Day 18 pi). Emerged nymphs sourced from eggs collected on this day were PCR and culture-positive for *R. felis*. Dogs C and D were culture-negative for *R. felis* throughout the experiment.

**Figure 1:**
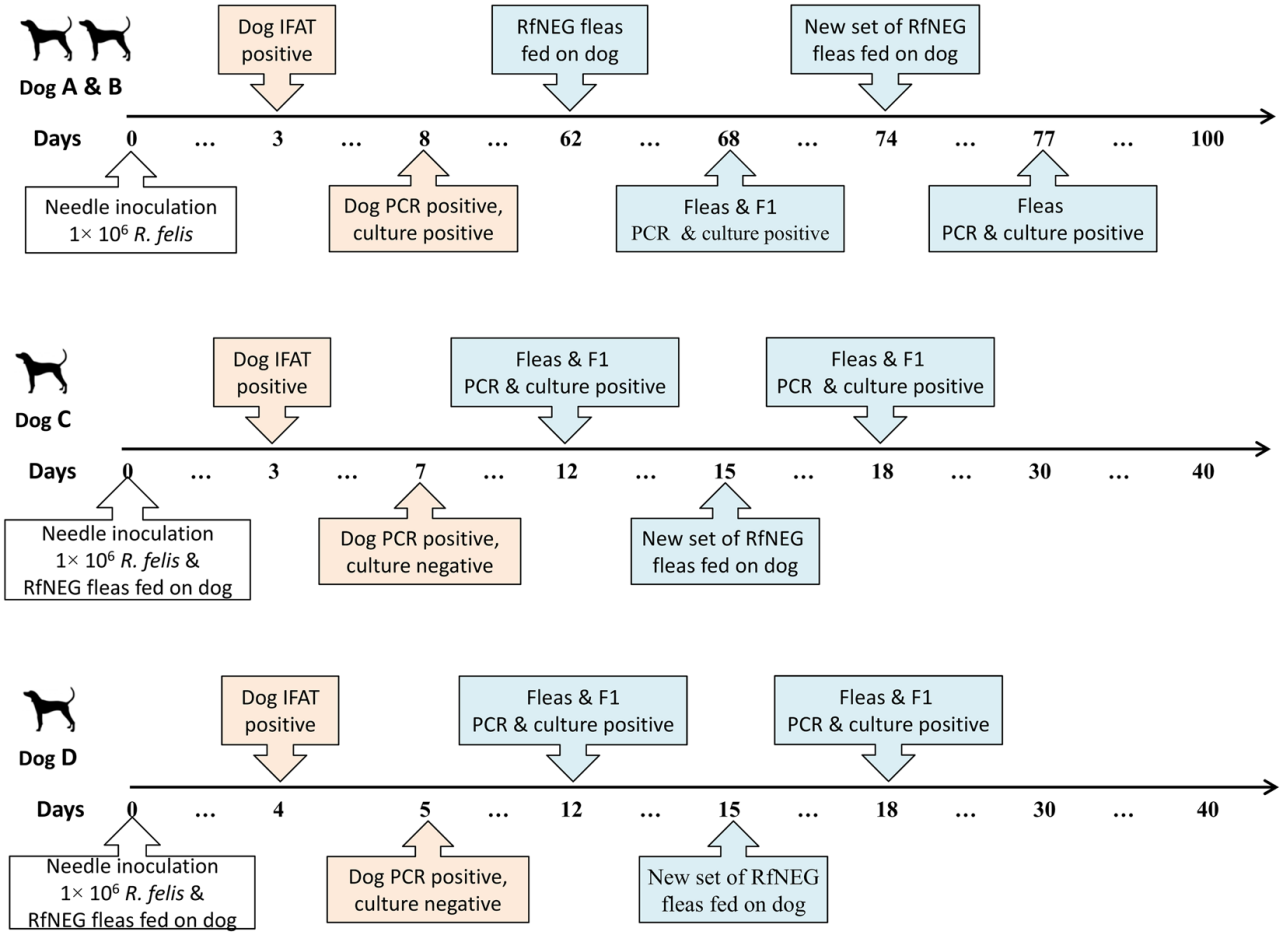
Flow diagrams showing the time lines of *R. felis* needle-inoculation experiment. The uncolored boxes indicate the beginning of experiment, the pink boxes report the initial IFAT, PCR and XTC-2 cell line culture results of the dogs and the light blue boxes report the PCR and XTC-2 cell line culture results of the fleas.

Dogs A, B, C and D were subsequently used to maintain RfPOS fleas for the following experiment. The time-line of the *R. felis* needle-inoculation experiment is shown in Fig. 1.

### Horizontal transmission of *R. felis* via co-feeding fleas

**Figure 2:**
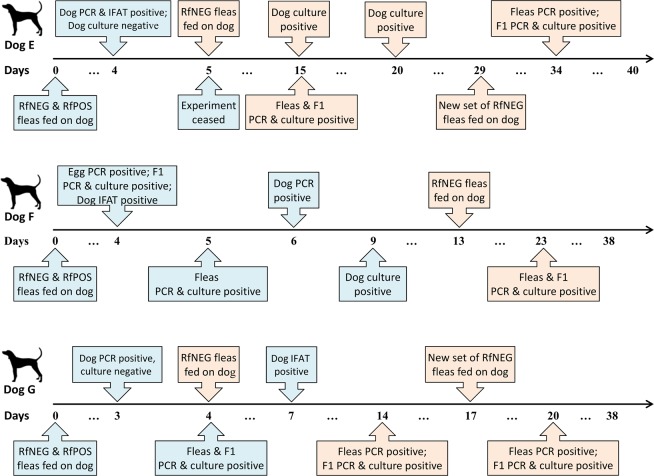
Flow diagrams showing the time lines of horizontal transmission of *R. felis*. The boxes in light blue indicate the horizontal transmission of *R. felis* via co-feeding fleas; the boxes in light pink indicate the horizontal transmission of *R. felis* via non-co-feeding fleas.

RfPOS and RfNEG fleas, each placed within separate feeding chambers were attached to opposite sides of Dogs E, F and G. Antibodies to *R. felis* were detected in Dogs E and F on day four, and in Dog G on day seven at titers of 1: 128, 1:256 and 1:256, respectively. *R. felis* was detected in Dog E by PCR on day four after the placement of RfPOS fleas. On day five, the experiment was ceased as the flea chamber containing the RfNEG fleas was compromised. Dog E remained negative by culture throughout the co-feeding experiment. Dogs F and G were RfPOS by PCR on days six and three after the placement of RfPOS fleas, respectively. Dog F was *R. felis* culture-positive on day nine, but Dog G was negative by culture throughout the experiment. Duplicate pools consisting of five RfNEG adult fleas placed on Dogs F and G became *R. felis* positive by PCR and culture on days five and four after RfPOS flea placement, respectively. Duplicate pools consisting of five emerged nymphs sourced from the eggs of adult fleas were also PCR- and culture- positive for *R. felis*. The time-lines for the horizontal transmission of *R. felis* via co-feeding are shown in Fig. 2.

### Horizontal transmission of *R. felis* via non-co-feeding fleas

All RfNEG and RfPOS flea chambers were removed from Dogs F and G on days five and four post-feeding, respectively and replaced with RfNEG fleas on days 13 and four, respectively from the commencement of the previous experiment. The RfNEG flea chamber was placed on Dog E on the same day the last experiment was compromised. RfNEG fleas placed on Dogs E, F and G tested in duplicate pools of five each, became *R. felis*-positive by PCR on day ten post feeding (on days 15, 23, and 14 RfPOS flea placement, respectively from the last experiment). All four pools of eggs collected from these RfPOS fleas were also *R. felis*-positive by PCR. Duplicate pools each consisting of five emerged nymphs sourced from these eggs were also PCR- and culture- positive for *R. felis*. Fleas sourced from Dogs E and F were also found *R. felis*-positive by culture.

**Figure 3:**
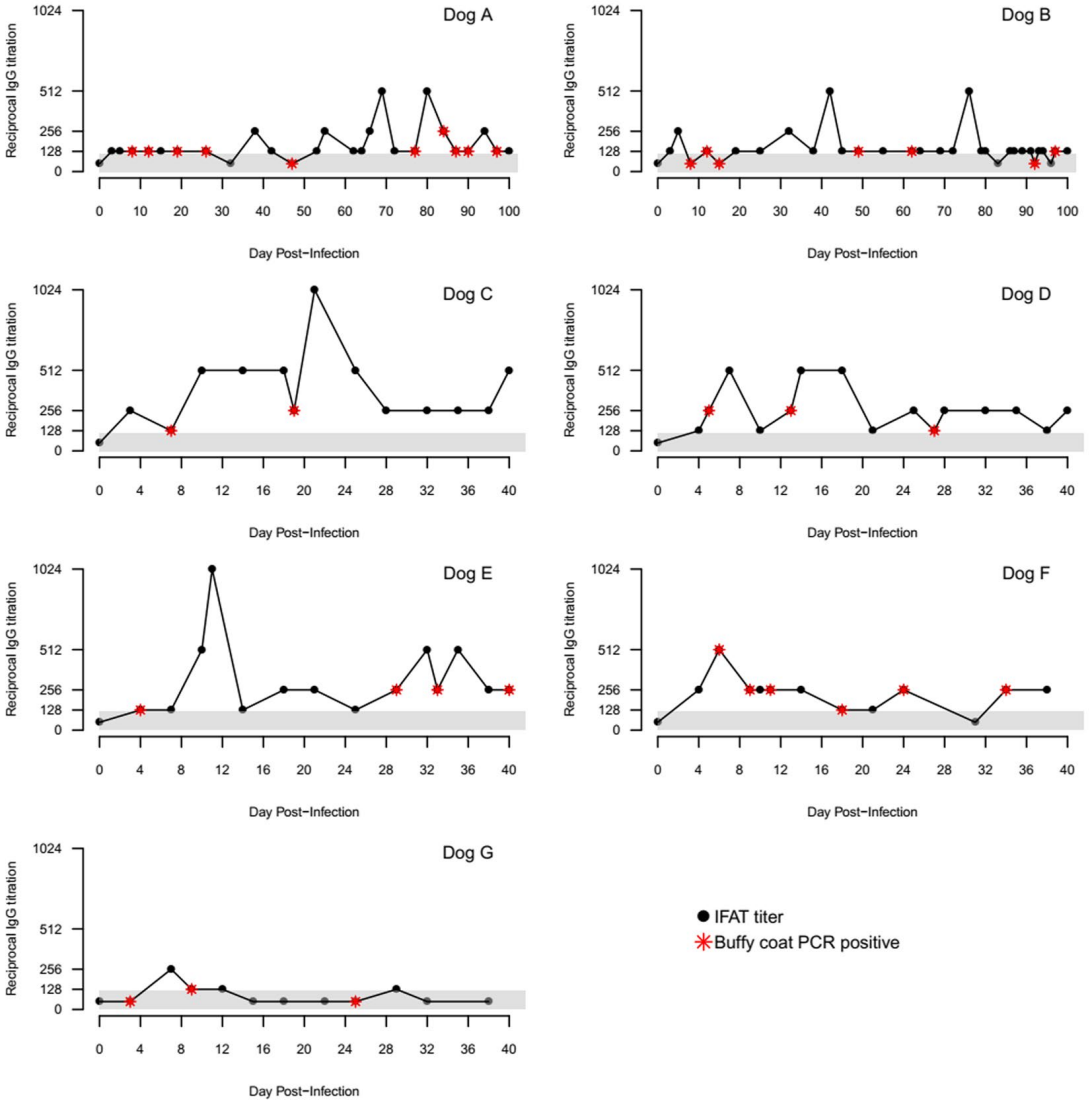
Graph showing the reciprocal IgG titers and PCR results for *R. felis* in blood of infected dogs by the days of post-infection. The shaded area represents seronegative titers of IFAT.

A repeat of feeding a new set of 40 RfNEG fleas placed on Dog E and G, resulted in the RfNEG fleas and egg pools tested becoming PCR-positive between 3–5 days post-feeding, or 20–34 days following original RfPOS flea placement. However*, R. felis* could not be isolated by culture in pools of these adult fleas. Duplicate pools each consisting of five emerged nymphs sourced from these eggs were PCR and culture positive for *R. felis*. Dogs F and G were negative by culture, but Dog E was *R. felis* positive by culture on days 15 and 20 of the original experiment. The time-line of horizontal transmission of *R. felis* via non-co-feeding fleas are shown in Fig. 2. Dogs E, F and G continued to remain PCR-positive for *R. felis* throughout the experiment (Fig. 3, Supplementary Fig. 3).

### Clinical signs

All dogs appeared healthy within the first five days of exposure to *R. felis*. Dogs A, B and F demonstrated mild, self-limiting diarrhea and reduced appetite lasting between 1–5 days. Dog B demonstrated mild self-limiting gingival petechial hemorrhages six days following experimental inoculation with *R. felis*, but this was not accompanied by any hematological abnormalities. None of the dogs were pyrexic throughout the study period.

### Hematological indices

**Figure 4:**
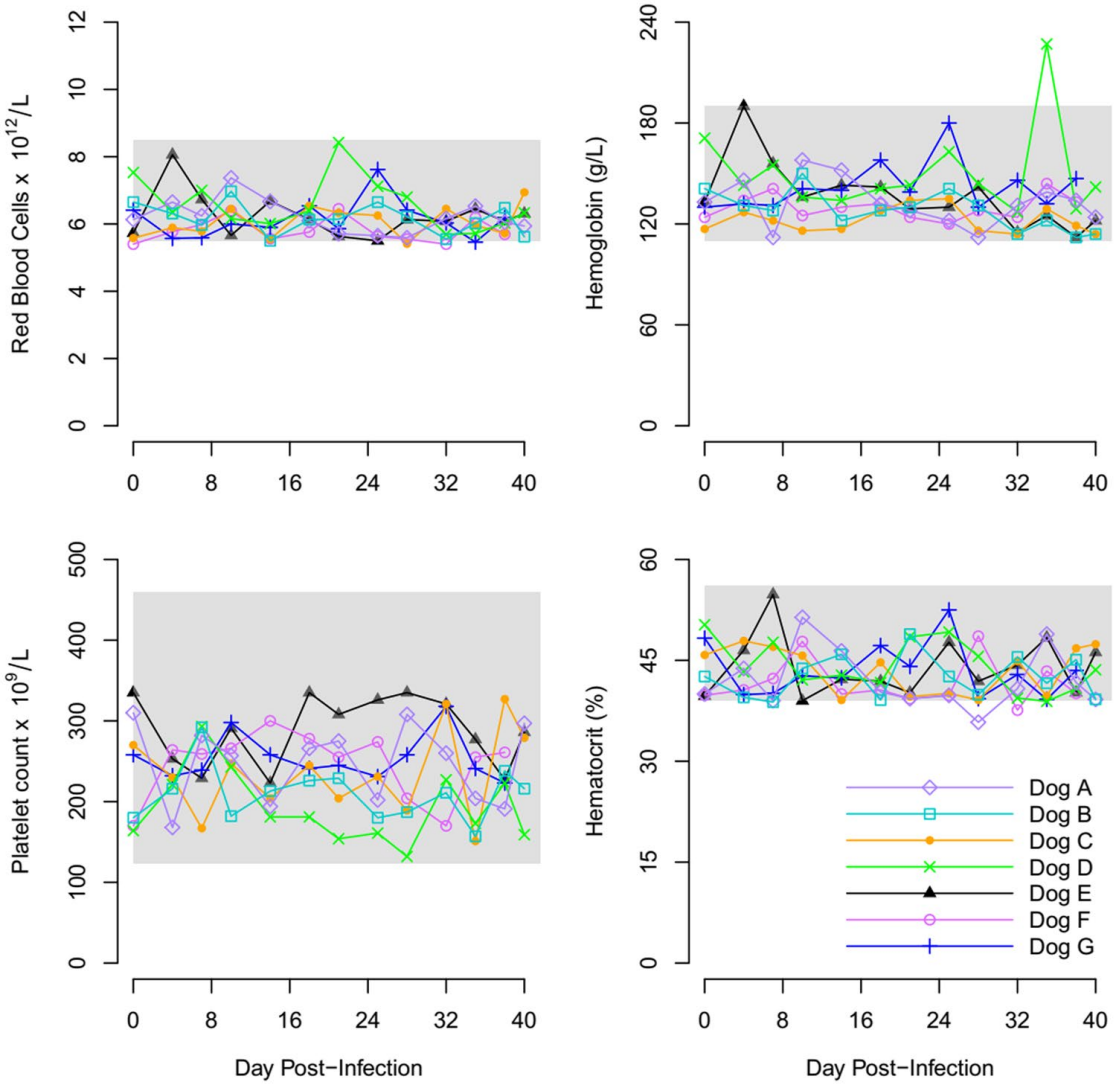
Graphs display the RBC, HGB, PLT and HCT of Dogs (**A–G**) during period of infection with *R. felis*. The shaded area represents the normal reference range for dogs.

**Figure 5:**
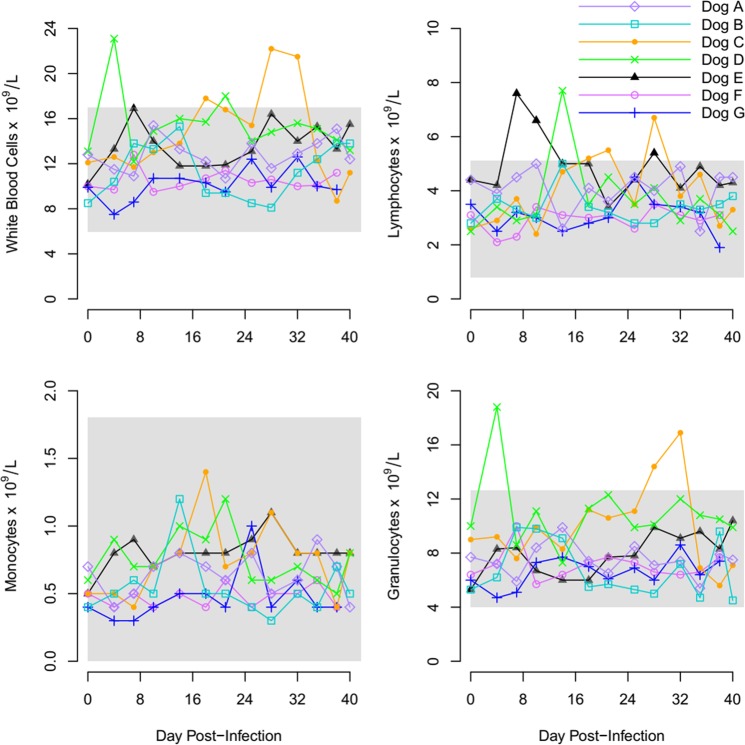
Graph showing the WBC, LYM, MON and GRAN during period of infection with *R. felis* of Dogs (**A–G**). The shaded area represents the normal reference range for dogs.

Hematological parameters for hematocrit (HCT), Red Blood Count (RBC), Hemoglobin (HGB) and Platelet count (PLT) remained within reference range for all seven experimental dogs (Fig. 4, Supplementary Fig. 4). The average white blood count (WBC), lymphocytes count (LYM), and granulocyte count (GRA) of the seven experimental dogs over the study period is displayed in Fig. 5 and Supplementary Fig. 5.

All the dogs were seronegative to *R. felis* prior to inclusion in the study. For 34/35 time points, *R. felis* DNA was detected in blood of dogs when they possessed low *R. felis* antibodies titres ≤1:256 (P < 0.001) (Fig. 3).

## Discussion

The results of this study demonstrate, for the first time, that dogs infected with *R. felis* are infectious to *C. felis felis* fleas following experimental inoculation with *R. felis* culture or bites of infected RfPOS fleas. We also demonstrate horizontal transmission of *R. felis* from naturally infected RfPOS fleas to dogs and in turn, to uninfected fleas feeding in the presence, as well as in the absence, of RfPOS fleas. Rickettsemias could be detected in the blood of naturally infected dogs by both PCR and culture between 3–6 days post-infection and the transmission of *R. felis* from dogs to RfNEG negative fleas between 3–10 days post-feeding. Rickettsemias were maintained for up to 100 days in experimentally inoculated dogs, until the study was ceased. Similarly, rickettsiaemia was intermittently detected in all naturally infected dogs’ blood throughout the experiment for up to 5 weeks following natural infection, or until the experiment was ceased (Fig. 3). These findings were consistent with a previous study that demonstrated *R. conorii* rickettsemia, the cause of the tick-borne zoonosis Mediterranean Spotted Fever, in their natural reservoir, the dog, for at least 4 weeks following the initial placement of *R. conorii*-positive *Rhipicephalus sanguineus* ticks^25^. Although *R. felis* can be maintained in cat fleas by vertical transmission without an infective blood source, the infection rate in *C. felis felis* declines from 63% to 2.5% after 12 generations^15^. This suggests that *R. felis* cannot be maintained in nature without the inclusion of an infective reservoir or amplifier vertebrate host.

All study dogs, regardless of the means of infection seroconverted to *R. felis* within a week post-infection. The IgG antibody titers of infected dogs were predominantly low and ranged from 1:128 to 1:1024. Not unexpectedly, rickettsemia was not detectable when antibody titers against *R. felis* were high (antibody titres ≥1: 512), except for Dog F (Fig. 3). A significant association was found between rickettsemia at time points when dogs displayed low antibody titres of ≤1:256. Nevertheless, uninfected fleas still acquired *R. felis* during a period of peak antibody titers, demonstrating that seropositive dogs may still serve as competent reservoirs and that the presence of antibodies may not elicit transmission-blocking abilities to the cat flea.

Uninfected fleas and their progeny acquired *R. felis* from both needle-inoculated or naturally infected dogs as early as 5 days post-feeding. In contrast, previous studies failed to demonstrate the vertical transmission of *R. felis* through artificial feeding chambers^26^. This study demonstrated that *R. felis* adults feeding on infected dogs can transmit the agent both vertically and transstadially, consistent with epidemiological role of a biological, vertebrate reservoir host.

Hirunkanokpun *et al*.^13^ demonstrated that the horizontal transmission from *R. felis* infected fleas to uninfected fleas in direct contact with each other can occur through the consumption of leftover *R. felis*-contaminated blood meals released by infected fleas or as a result of mating. Similarly, the transmission of *R. felis* through direct co-feeding of numerous arthropods such as *Ixodes scapularis*, *Xenopsylla cheopis* and mosquitoes has been demonstrated^11,27–29^. However, the role of *C. felis felis* is more strongly supported through the identification of *R. felis* in the salivary glands of the cat flea^30^. Our study significantly strengthens the biological role of *C. felis felis* as the natural biological vector of *R. felis* by demonstrating horizontal, transstadial and vertical transmission of *R. felis* between infected and uninfected cat fleas feeding on dogs that are not co-feeding on the same host or artificial media or in direct contact.

Our study is also the first to successfully isolate *R. felis* from the blood of vertebrae hosts, including the dog. Molecular assays have been widely used for detection of *Rickettsia* DNA but these assays do not distinguish viable and nonviable agents. The isolation of the agent from the blood of dogs in culture demonstrates the definitive viability of the rickettsemia.

Typically, a reservoir host of a vector-borne agent will not demonstrate overt clinical signs (i.e. remain asymptomatically infected). In our study, Dogs A, B and F demonstrated mild, self-limiting diarrhea and reduced appetite that was not associated with rickettsemia or hematological abnormalities. A single dog (Dog B) demonstrated mild self-limiting petechial hemorrhages of its gums six days following experimental inoculation with *R. felis*, but this too, was not accompanied by any hematological abnormalities. Nevertheless, none of the dogs were found pyrexic throughout the study period. The absence of severe clinical signs in infected dogs potentially suggests the ecological coadaptation and reservoir role of domestic dogs for *R. felis*^23^.

Hematological parameters of the study dogs were also predominantly normal. Dogs C and D, both experimentally inoculated, developed a mild leukocytosis owing to neutrophilia and mild transient lymphocytosis but this did not necessarily correspond to periods of rickettsemia. Dog E, naturally infected developed mild lymphocytosis on days 8–12 pi. Other than these mild abnormalities, all hematological parameters of the study dogs remained normal. These observations are in stark contrast to dogs infected with *Rickettsia rickettsii*, the agent of Rocky Mountain Spotted Fever, that develop lymphopenia, pyrexia, decreased appetite and petechia^31^. Due to the largely subclinical nature of infection, our results support that dog can act as a natural reservoir host.

In conclusion, this research provides unequivocal evidence that domestic dogs can act as natural vertebrate reservoir hosts for *R. felis* URRWXCal2. Unlike most rickettsial zoonoses that are sylvatic in nature, the ability of up to 10% of dog populations in the Asia Pacific to harbor circulating rickettsemias Hii *et al*.^23^, coupled with the close association between people and domestic dogs and their fleas, brings this emerging yet poorly recognized zoonosis Teoh *et al*.^32^, closer to home. Veterinarians have an important role in advocating flea control in domestic pets and educating clients on the risks of flea exposure to themselves and their families.

## Methods

### Ethics approval

Ethics approval for this study was granted through Tay Nguyen University Animal Ethics Committee (ID: KCNTY-012017). All methods were approved by Animal Ethics Committee of Tay Nguyen University and were carried out in accordance with the approved guidelines.

### Research dogs

Two pregnant mixed-breed dogs were recruited from a registered breeder at four weeks of gestation. While pregnant, both dogs were housed in a clean, flea-free indoor environment and administered a combination topical treatment of imidacloprid and permethrin (Advantix^®^, Bayer) and imidacloprid and moxidectin (Advocate^*®*^, Bayer) on a monthly basis till eight weeks post-partum. All puppies born to the dams were dewormed at 2, 4, 6, 8 and 12 weeks of age with pyrantel (Drontal^®^ Puppy, Bayer) and monthly thereafter. The puppies were also administered a single dose of Advantix^®^ (Bayer) at 7 weeks of age. All puppies were vaccinated with CanigenDH (A2) PPI/L (Virbac) and rabies at 12 weeks of age. At this time, nine puppies were introduced into the research facility animal house and placed in individual concrete kennels surrounded by water moats and raised on commercial dry food. Two weeks prior to commencing the experiment all pups were subject to a full health screen and tested for antibodies to spotted fever and typhus group rickettsiae by microimmunofluorescence antibody testing (IFAT) and the presence of SFG rickettsial DNA by PCR prior to inclusion in the study at 14 weeks of age. Following conclusion of the study, all dogs were treated with doxycycline 10 mg/kg bid for 2 weeks prior to being re-homed.

### Source of *R. felis*-negative fleas

*R. felis*-PCR negative cat fleas (*Ctenocephalides felis felis*) were collected from community cats that were seronegative by IFAT as well as negative by PCR for SFG rickettsiae targeting the *omp*B gene^33^. In total, 100 collected fleas were maintained within feeding chambers attached to a shaved area on either side of the abdomen or chest of two *R. felis* naïve 14-week old puppies, Dogs H and I. Eggs were collected from each feeding chamber over a period of one week and a subset consisting of two pools of five eggs each were tested for *R. felis* DNA from each dog using PCR. Once confirmed as negative, flea isolates were further propagated to allow emerged nymphs to continually be maintained on these dogs (see Flea breeding section). Two pools, each consisting of ten adult fleas were subjected to DNA extraction and tested by PCR prior to each experimental placement on study dogs, to confirm the continued absence of *R. felis* DNA within the population of RfNEG propagated fleas.

### Source of *R. felis* positive fleas

RPOS fleas for this study were sourced through the experiment of *R. felis* needle-inoculation (see below).

### *R. felis* needle-inoculation of dogs

*R. felis* was cultured in XTC-2 cell lines following previously described protocols^34,35^. Dogs A, B, C and D were inoculated with 1 × 10^6^ *R. felis* suspended in 2 mL of 0.9% sodium chloride, between the shoulder blades by subcutaneous injection on Day 0. Fifty fleas (40% male and 60% female) were placed in each feeding chamber attached to a shaved area on either side of the abdomen or chest, away from the inoculation site, on day one pi of Dogs C and D, and on day 62 of Dogs A and B. Eggs together with five adult fleas were collected from chambers daily. Duplicate pools of five eggs each, along with a single pool of five adult fleas were tested for *R. felis* DNA by PCR daily, together with two pools of five newly emerged nymphs. This was repeated on adult fleas, eggs and their newly emerged nymphs on the day RfNEG fleas became positive to *R. felis*. A duplicate pool of five fleas and five nymphs were also subject to *R. felis* culture once RfNEG fleas became positive to *R. felis* by PCR (see Cell culture section). Following this, Dogs A, B, C and D were used for the maintenance of *R. felis* positive (RfPOS) fleas.

### Horizontal transmission of *R. felis* via co-feeding fleas

This experiment aimed to investigate if *R. felis* can be transmitted horizontally from RfPOS to RfNEG fleas when co-feeding on dogs. Approximately 40 RfPOS and 40 RfNEG fleas with a sex ratio of 40% male and 60% female were loaded onto separate feeding chambers and placed on opposite sides of the chest or abdomen of Dogs E, F and G. Eggs together with five RfNEG adult fleas were collected from feeding chambers daily. Single pools of five RfNEG fleas and duplicate pools of five eggs were tested for *R. felis* DNA by PCR daily until RfNEG fleas became PCR positive. DNA extraction and PCR testing was repeated on adult fleas, eggs and duplicate pools of five corresponding newly emerged nymphs on the day RfNEG fleas became positive to *R. felis*. A duplicate pool of five remaining fleas and five nymphs were also subject to *R. felis* culture once RfNEG fleas became positive to *R. felis* by PCR (see Cell culture section).

### Horizontal transmission of *R. felis* from non-co-feeding RfPOS to RfNEG fleas

**Figure 6:**
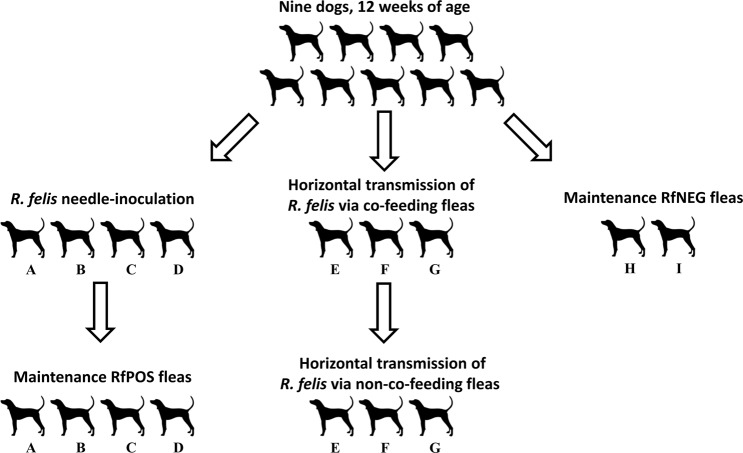
Diagram showing the study dogs allocated for study.

This experiment aimed to investigate if *R. felis* can be transmitted horizontally from RfPOS to RfNEG fleas in the absence of co-feeding on dogs. Fifty RfNEG fleas in the same sex ratio were loaded onto flea chambers and placed on the Dogs E, F and G following removal of all RfPOS flea chambers. New sets of RfNEG fleas were placed on these dogs at weekly intervals until *R. felis* was detected in RfNEG flea pools and their eggs using PCR. Testing was carried out as per the co-feeding experiment. Similarly, once PCR positive, RfNEG fleas and newly emerged nymphs were then subject to *R. felis* culture. The overview of allocation of dogs for this study is shown in Fig. 6.

### Clinical examination and sample collection

Experimental dogs were subject to daily physical examinations. Between 2–5 mL of whole blood was collected into EDTA and plain tubes over the course of the study. The EDTA blood was subjected to i) complete blood counts bi-weekly, ii) DNA extraction and PCR performed daily until rickettsemia observed and bi-weekly after that, iii) inoculation of XTC-2 cell lines once weekly. Sera were shipped to the Australian Rickettsial Reference Laboratory (ARRL), Geelong, Victoria, Australia for the detection of antibodies titers against *R. felis* using IFAT.

### Flea breeding

To maintain the strains of RfNEG and RfPOS fleas, fifty fleas in the same sex ratio were placed within feeding chambers and attached via stretchable wrap to shaved areas of the chest or abdomen of dogs and replaced weekly with a new set of fleas. Flea eggs were collected from the flea chambers on a daily basis and propagated according to the protocol reported by Rust *et al*.^36^. Duplicate pools, each consisting of ten eggs and ten emerged adult fleas were subjected to DNA extraction and PCR for the presence of *R. felis* (see *R. felis* PCR section).

### Cell culture

*R. felis*-infected and uninfected XTC-2 cell lines for this study were kindly provided by the ARRL. *R. felis* cultures performed according to Hii *et al*.^34^.

### Isolation of *R. felis* from fleas and dog blood

The isolation of *R. felis* into the XTC-2 cell lines from cat fleas and buffy coat of dogs was performed following a previously described protocols^34,37^.

### R. felis PCR

Genomic DNA from buffy coat was extracted using QIAamp^®^ DNA Blood Mini Kit (QIAGEN, Hilden, Germany) according to manufacturer’s instructions. Genomic DNA from XTC-2 cell lines, fleas and eggs were extracted using the DNeasy^®^ blood & Tissue Kit (QIAGEN, Hilden, Germany) in accordance with the manufacturer’s instructions. The extracted DNA was subjected to conventional PCR, using the primers ompB-F 5′-CGACGTTAACGGTTTCTCATTCT-3′ and ompB-R 5′-ACCGGTTTCTTTGTAGTTTTCGTC-3′ targeting the partial *omp*B gene^23,33^. Real-time PCR (qPCR) using the primers CS-F (5′-TCGCAAATGTTCACGGTACTTT-3′) and CS-R (5′-TCGTGCATTTCTTTCCATTGTG-3′), and the probe CS-P (5′-6-FAM-TGCAATAGCAAGAACCGTAGGCTGGATG-BHQ-1-3′) targeting the partial *glt*A gene was used to estimate the *R. felis* concentration in the XCT-2 cell lines^38^.

### Immunofluorescence antibody testing

An IFAT was performed at ARRL following a previously described protocol^34,39^. Readings were repeated by a second independent observer, with a third independent observer recruited to resolve any discrepancies.

### Blood count

Complete blood counts were carried out using the BC-2800Vet Auto Hematology Analyzer (Mindray, Shenzhen, China).

### Statistical analysis

Statistical analyses were performed using R^40^. The data were analyzed using descriptive statistic.

### Reporting summary

Further information on research design is available in the Nature Research Reporting Summary linked to this article

## Supplementary information

Dataset 1.

Dataset 2.

Dataset 3.

## Data Availability

Data supporting the findings of this study are available within the main text. All data are available from the corresponding author upon request.
